# The Bioactive Compounds and Antioxidant Capacity of Nopal Cladodes (*Opuntia* spp.) as Influenced by Irrigation

**DOI:** 10.3390/antiox15070787

**Published:** 2026-06-24

**Authors:** Edén A. Luna-Zapién, Jorge A. Zegbe, Andrea de J. Campos-Badillo, Jolanta E. Marszalek, Juan R. Esparza-Rivera, Jorge A. Meza-Velázquez

**Affiliations:** 1 Facultad de Ciencias Químicas, Universidad Juárez del Estado de Durango, Av. Artículo 123 s/n, Gómez Palacio C.P. 35010, Durango, Mexico; edenareli.luna@ujed.mx (E.A.L.-Z.); juan.esparza@ujed.mx (J.R.E.-R.); 2 Instituto Nacional de Investigaciones Forestales, Agrícolas y Pecuarias, Campo Experimental Pabellón, km 32.5 Carretera Aguascalientes-Zacatecas, Pabellón de Arteaga C.P. 20670, Aguascalientes, Mexico; 3 Facultad de Ciencias Biológicas, Universidad Autónoma de Coahuila, Carretera Torreón-Matamoros km 7.5, Torreón C.P. 27276, Coahuila, Mexico; andreacampos@uadec.edu.mx (A.d.J.C.-B.); j.marszalek@uadec.edu.mx (J.E.M.)

**Keywords:** antioxidants, cladodes, polyphenolic compounds, water deficit, flavonoids, supplemental irrigation

## Abstract

The prickly pear is a crop of socioeconomic relevance in arid regions, and its productivity and chemical composition depend on water availability. The effect of irrigation on the crop’s biochemical quality was evaluated. Cladodes of cultivars: ‘Amarilla Olorosa’, ‘Cristalina’, ‘Dalia Roja’, and ‘Roja Lisa’, were subjected to three treatments: no irrigation (NI), supplemental irrigation (SI), equivalent to 50% of the crop’s evapotranspiration, and full irrigation (FI). Subsequently, cladodes were collected, and total polyphenols and flavonoids, polyphenol profile, and antioxidant capacity were determined. Cladodes under NI had the highest concentrations of flavonoids, although the lowest values of total polyphenols. In the cladode extracts, myricetin, rutin, catechin, as well as caffeic, chlorogenic, dihydroxybenzoic, and vanillic acids were identified. Overall, cladodes grown under FI and SI showed higher levels of phenolic acids (caffeic, chlorogenic, and vanillic), while concentrations of catechin, myricetin, and rutin were higher under SI and NI. Antioxidant capacity was higher in NI cladodes assessed by ABTS and DPPH, while the FRAP assay showed higher values under SI. Among the cultivars, ‘Amarilla Olorosa’ stood out for its high content of bioactive compounds, confirming the potential of nopal cladodes as a source of antioxidant metabolites with agro-industrial applications.

## 1. Introduction

Climate change, characterized by global warming, has significantly affected agriculture in arid and semi-arid regions [1]. These areas experience extreme temperature increases, increased soil erosion [2], and, consequently, water scarcity. The lack of water has significantly limited agricultural practices in arid and semi-arid regions [3]. Therefore, alternatives for efficient water management, such as supplemental irrigation (SI), have been investigated for the production of various crops [4]. SI is an irrigation method in which additional water is applied to the crop when rainfall is insufficient to mitigate the adverse effects of drought [5]. For fodder crops grown in arid and semi-arid regions, this type of irrigation is an important strategy that yields results comparable to full irrigation but higher than under dry conditions [6,7,8]. Although research on SI effects on the levels of bioactive compounds in plants is limited, for example, SI treatment has increased polyphenol content and antioxidant activity in sorghum [9] and tomatoes [10].

In regions with limited irrigation water, cultivation of *Opuntia* species is widespread. These plants (prickly pear, nopal) have anatomical and morphophysiological traits that confer adaptation to dry environments, including high resilience to climate change, high water-use efficiency, and high biomass production [2]. They are rich in fiber (soluble and insoluble), vitamins, and minerals [11]. They also contain secondary metabolites, which plants produce as part of their defense mechanisms, and these are considered bioactive compounds that offer health benefits for consumers. It has been reported that prickly pear contains a wide diversity of these compounds, with a particular prevalence of phenolic acids and flavonoids, such as gallic acid, chlorogenic acid, dihydroquercetin, quercetin, rutin, isorhamnetin, and kaempferol [11,12]. These compounds are known for their potent antioxidant activity and significant benefits in the prevention and treatment of chronic and degenerative diseases, including cancer, hypertension, obesity, and cardiovascular diseases [13]. At the same time, the qualitative and quantitative profiles of polyphenolic compounds in plants vary with maturity stage, cultivar, soil type, and nutritional status [14,15].

Previous studies within the same experimental framework have shown that irrigation management markedly affects plant water status, fruit development, yield, fruit quality, postharvest performance, and the storability of *Opuntia* spp. Cultivars [16,17,18]. These findings highlighted the agronomic and commercial importance of supplemental irrigation strategies in cactus pear cultivation. Despite the significance of irrigation management, there is limited information on its effects on the phytochemical composition and antioxidant capacity of mature nopal cladodes.

In arid regions, the prickly pear is valued as a nutritious food source. Young, fresh nopal cladodes, “nopalitos,” are consumed in Mexico and the United States [11]. However, cladodes at advanced stages of maturity (>5 months) are not used in the human diet because of their undesirable texture, attributed to high levels of insoluble fiber [19]. These older cactus parts are generally used as cattle fodder [11]. For that reason, in specialized prickly pear orchards, severe pruning is applied to stimulate the yield of fresh growth and fruit [20]. This practice generates 6 to 10 tons of cladode waste per hectare annually, posing an environmental issue and incurring additional disposal costs [21]. However, this pruning waste could also be used by the pharmaceutical industry as a powder source of bioactive compounds for human health, nutrition, and disease, as noted by El-Mostafa et al. [22]. Therefore, this research aims to assess the influence of irrigation on bioactive compounds in mature prickly pear cladodes, with the goal of identifying future applications. It was hypothesized that bioactive compounds in mature cladodes would be differentially modified by the interaction between irrigation treatments and prickly pear cultivars.

## 2. Materials and Methods

### 2.1. Experimental Site

The experiment was conducted at the Zacatecas Experimental Field in Calera, Zacatecas, Mexico (latitude 22°54′ N, longitude 102°39′ W, elevation 2197 m). The site has an average annual temperature of 14.6 °C and receives 416 mm of annual precipitation; the highest rainfall (75%) occurs in July and October. The average annual evaporation is 1.609 mm. The orchard soil has a loamy texture, 1.73% organic matter, and a pH of 7.75.

Plant material and experimental design

Mature nopal plants (thirteen years old) were used in the present study. The cultivars used were ‘Cristalina’ (*Opuntia albicarpa* Scheinvar), ‘Amarilla Olorosa’ (*Opuntia* spp.), ‘Roja Lisa’ (*O. ficus-indica* (L.) Mill), and ‘Dalia Roja’ (*O*. spp.). These plants were exposed to varying amounts of water from February to May across three consecutive growing seasons (2017–2019). For this, the plots were divided into three lots per cultivar. The first lot received no additional water other than rainfall and was designated as no irrigation (NI). The second received water when the soil water content (θ) was reduced to 50% and was labeled as supplementary irrigation (SI). The last received water weekly, supplying 100% of the crop’s water evapotranspiration, and was defined as full irrigation (FI). The irrigation schedule was estimated using a weekly soil water balance, with θ at field capacity (θ_CC_ = 0.28 cm^3^ cm^−3^) and θ before each irrigation (θ_AR_) measured at a depth of 20 cm.

The experiment was conducted using a divided plot design in a randomized complete block arrangement, with four replications. The experimental unit comprised 9 plants, and 5 kg of pest-free, mechanically undamaged cladodes were collected from each treatment. After collection, the cladodes were cleaned, the spines were manually removed, and the plant matter was sanitized. Finally, the samples were freeze-dried using a LABCONCO FreeZone Triad Cascade Benchtop freeze dryer (USA) for five days at a collector temperature of −80 °C and a vacuum pressure of 0.040 mBar. Moisture and dry matter contents of cladodes were determined before and after freeze-drying and are presented in Supplementary Material (Table S1).

### 2.2. Chemical Reactants

Ethyl alcohol, sodium carbonate, potassium ferricyanide, ferric chloride, sodium nitrite, and sodium hydroxide were supplied by J.T. Baker (Phillipsburg, NJ, USA). The reagents: Folin-Ciocalteau, potassium persulfate, dibasic potassium phosphate, trichloroacetic acid, phosphoric acid, aluminum trichloride, ABTS (2,2′–azino-bis(3-ethylbenzothiazolin–6–sulfonic acid)), DPPH (1,1-diphenyl-2-picrylhydrazyl), Trolox (6-hydroxy-2,5,7,8-tetramethylcroman-2-carboxylic acid), the HPLC-grade solvents: water, methanol, and acetonitrile; and the HPLC standards: gallic acid, chlorogenic acid, syringic acid, caffeic acid, dihydroxybenzoic acids, catechin, quercetin, rutin, and myricetin were acquired from Sigma-Aldrich (St. Louis, MI, USA).

### 2.3. Preparation of Methanolic Extracts

Exactly 0.25 g of freeze-dried nopal powder and 5 mL of methanol were homogenized using an Ultraturrax IKA T25D (IKA^®^ Works, Inc., Wilmington, DE, USA) at 13,500 rpm for 1 min. The mixture was then magnetically shaken for 16 h at 4 °C in the dark. The mixture was centrifuged at 3800 *g* for 15 min, and the supernatant was collected and stored at 4 °C in the dark for no longer than 7 days prior to analysis. All extractions were performed in triplicate [23]. These extracts were used to determine the total phenolic content, total flavonoids, and antioxidant activity.

### 2.4. Total Polyphenols Content, TPC

The Folin–Ciocalteu method was used for TPC analysis. 100 μL of nopal cladode extract, 950 μL of DI water, and 50 μL of Folin–Ciocalteu reagent were vortexed. After 5 min, 800 μL of Na_2_CO_3_ (7.5% *w*/*v*) was added, and the mixture was vortexed again. The mixture was incubated for 30 min in the dark at 25 °C. The absorbance of the solution was measured at 745 nm using a UV-Vis spectrophotometer (HACH DR 5000). The total phenolic content was quantified using a gallic acid standard curve (25–200 µg/mL; R^2^ = 0.99).

The results were expressed as mg of GAE per g of dry weight (dw). The analyses were performed in triplicate [24].

### 2.5. Total Flavonoids

Exactly 250 μL of methanolic extract of nopal cladodes, 1.25 mL of distilled water, 75 μL of 5% NaNO_2_, and 150 μL of 10% AlCl_3_ · 6H_2_O were mixed and allowed to stand for 5 min. Then, 500 μL of 0.1 M NaOH was added, and the volume was adjusted to 2.5 mL with distilled water. The sample was incubated for 30 min and analyzed at 510 nm using a UV-Vis spectrophotometer (HACH DR 5000). The total flavonoid content was determined using a catechin calibration curve (25–400 µg/mL; R^2^ = 0.99). Results were expressed as milligrams of catechin equivalents per g of dry matter. Analyses were performed in triplicate [25].

### 2.6. Identification and Quantification of Polyphenolic Compounds by HPLC-DAD

Chromatographic analysis was performed using an Agilent 1200 HPLC (Agilent Technology, Palo Alto, CA, USA) equipped with a DAD, a quaternary pump, and two LiChrospher C18 columns (5 μm, 4 mm × 150 mm, Phenomenex) connected in series. The temperature and flow rate were 25 °C and 0.5 mL/min, respectively. A 20 μL sample (methanolic extract), previously filtered through 0.45 μm PTFE filters, was injected. The mobile phase consisted of (A) 0.3% phosphoric acid, (B) acetonitrile, and (C) methanol. The gradient was 95% (A), 2% (B), and 3% (C) up to 15 min; 60% (A), 15% (B), and 25% (C) up to 20 min; 62% (A), 18% (B), and 20% (C) up to 25 min; 60% (A), 20% (B), and 20% (C) up to 35 min; 58% (A), 22% (B), and 20% (C) up to 45 min; 55% (A), 25% (B), and 20% (C) up to 50 min; and 95% (A), 2% (B), and 3% (C) up to 60 min. Phenolic compounds were analyzed at three wavelengths: 280 nm, 316 nm, and 365 nm. Standards of caffeic acid, chlorogenic acid, dihydroxybenzoic acid, vanillic acid, catechin, myricetin, and rutin were used to identify and quantify phenolic compounds [26]. External calibration curves were prepared for chlorogenic acid, caffeic acid, dihydroxybenzoic acid, vanillic acid, catechin, myricetin, and rutin. Details of the calibration ranges, regression equations, and coefficients of determination (R^2^) used for quantification are provided in Supplementary Material (Table S2).

### 2.7. Evaluation of the Antioxidant Capacity

The effect of different irrigation regimes on the antioxidant capacity of 4 nopal cultivars was also tested. The (2,2′-azino-bis-(3-ethylbenzothiazoline-6-sulfonic) acid) (ABTS), 2,2-diphenyl-1-picrylhydrazyl (DPPH), and ferric reducing antioxidant power (FRAP) assays were used as described by González-Centeno et al. [24]. For the first assay, a 7 mM ABTS solution was prepared and mixed with a 2.6 mM sodium persulfate solution. The mixture was incubated for 24 h in the dark. Then, 50 μL of the extract was mixed with 1950 μL of the ABTS solution. The decrease in absorbance was measured at 734 nm using a UV-Vis spectrophotometer (HACH DR 5000). For DPPH analysis, 50 μL of extract and 1950 mL of 2,2-diphenyl-1-picrylhydrazyl (DPPH) solution were mixed and incubated for 1 h, and the absorbance was measured at 515 nm using a UV-Vis spectrophotometer (HACH DR 5000) [27]. Likewise, for the Ferric Reducing Antioxidant Power (FRAP) assay, a solution of 0.01 M TPTZ (2,4,6-tripyridyl-S-triazine), 0.02 M ferric chloride hexahydrate, and 300 mM sodium acetate in 0.01 M HCl was prepared at pH 3.6. A total of 50 μL of the extract and 1950 μL of the FRAP reagent were mixed, incubated for 30 min, and the absorbance was read at 593 nm using a UV-Vis spectrophotometer (HACH DR 5000) [28]. Antioxidant capacity was calculated using a standard curve with Trolox as the standard (R^2^ = 0.99). The results were expressed as milliequivalents of Trolox per g of dry weight (dw). The analysis was performed in triplicate.

### 2.8. Statistical Analysis

The data were analyzed using a randomized complete block model, and mean comparisons were performed using Fisher’s least significant difference test at *p* ≤ 0.05. Pearson correlation analysis was used to evaluate the relationships among total polyphenol content, total flavonoid content, and antioxidant capacity. All calculations were performed using STATISTICA^®^ 7.0 (StatSoft, Inc., Tulsa, OK, USA).

## 3. Results and Discussion

### 3.1. Total Polyphenols

**Figure 1:**
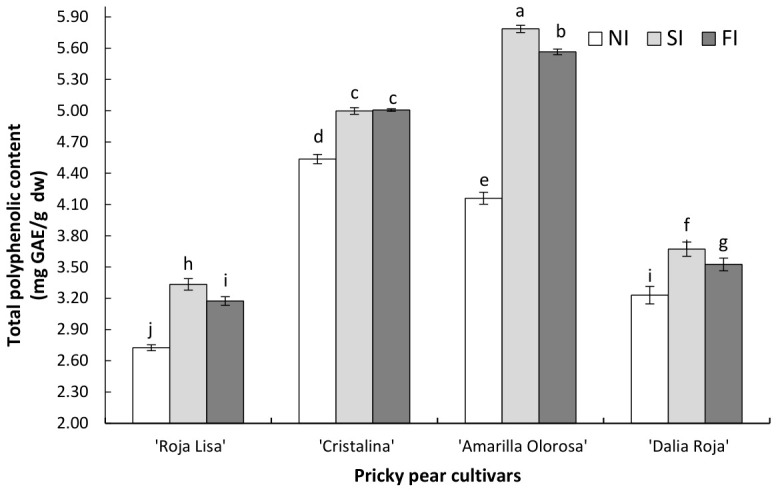
Total polyphenolic content (mg gallic GAE/g dw) of prickly pear cladodes subjected to irrigation treatments: no irrigation (NI), supplemental irrigation (SI), and full irrigation (FI) (*n* = 3). Differences between means were assessed using Fisher’s least significant difference test at *p* ≤ 0.05. Mean values (±standard deviation) followed by different letters are statistically different. Vertical bars represent the standard deviation of the means.

The total polyphenolic content on a dry weight (dw) basis (TPC, mg GAE/g dw) of cladodes from different cultivars under irrigation treatments is shown in Figure 1.

In general, the cladodes of prickly pear cultivars under SI had the highest TPC. The ‘Amarilla Olorosa’ cultivar yielded the highest TPC under SI (~5.8 ± 0.08 mg GAE/g dw). Interestingly, all nopales subjected to water stress (NI) had the lowest TPC values, with the ‘Roja Lisa’ cultivar the lowest (~2.7 ± 0.31 mg GAE/g dw). Similar behavior has been reported in poplar tree species [29], *Eucalyptus globulus* Labill and *Eucalyptus viminalis* Labill [30], and *Rehmannia glutinosa* [31], where polyphenolic compound content decreased under severe water-deficient episodes.

In general, partial water stress increases TPC. However, when plants are subjected to severe water deficit, polyphenolic compounds decrease. According to Bettaieb et al. [32], severe water stress can affect enzymes involved in the biosynthesis of phenolic compounds, leading to partial inactivation and reduced polyphenolic content under harsh water-limiting conditions. Also, variations in the content and types of secondary metabolites are mediated by each species’ genotype in response to the same type of stress. Plants of different genotypes of the same species under stress are likely to increase secondary metabolic activity; however, the pathways that produce these compounds differ, resulting in unique secondary metabolite profiles [33,34].

### 3.2. Total Flavonoids

**Figure 2:**
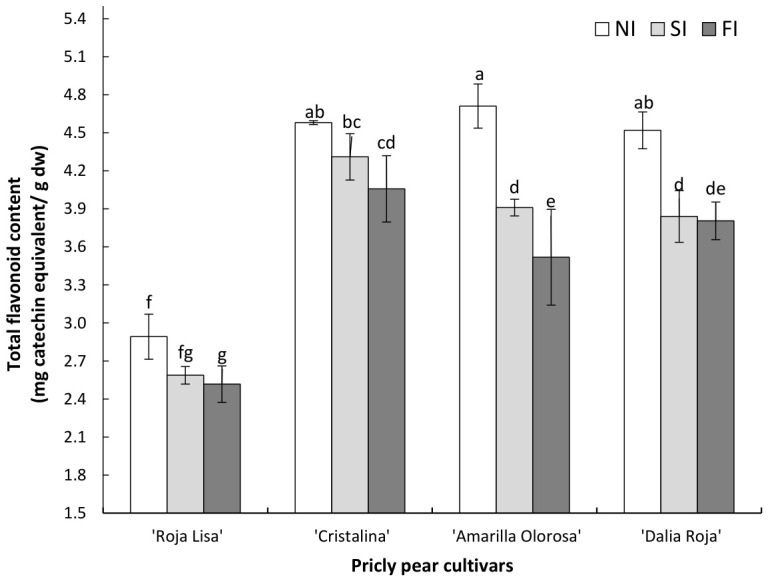
Total flavonoid content (mg eq. catechin/g bs) of prickly pear cladodes undergoing irrigation treatments: no irrigation (NI), supplemental irrigation (SI), and full irrigation (FI) (n = 3). Differences between means were assessed using Fisher’s least significant difference test at *p* ≤ 0.05. Mean values (±standard deviation) followed by different letters are statistically different. Vertical bars represent the standard deviation of the means.

The total flavonoid content of cladode extracts from prickly pear cultivars under three irrigation regimes is shown in Figure 2. Overall, plants under NI had the highest levels of these compounds (~3662 ± 0.74 mg catechin eq./g dw). However, ‘Roja Lisa’ cladodes yielded the lowest flavonoid levels across all irrigation regimes compared with the other cladode cultivars.

Several studies have reported increased flavonoid content, for instance, in tomato fruit [10], wheat leaves [35], and canola plants [36] under water-scarce conditions. These findings suggest that flavonoids may play a protective role when plants are exposed to water deficit (NI and SI). During drought stress, plants can experience oxidative stress, leading to the buildup of reactive oxygen species (ROS) that can damage cellular structures and impair plant growth and development [37]. Flavonoids, due to their antioxidant properties, can reduce ROS levels, which helps lower oxidative stress [35].

### 3.3. Identification and Quantification of Polyphenolic Compounds by HPLC-DAD

Methanolic extracts of cladodes from four prickly pear cultivars under irrigation treatments were evaluated for the presence and quantity of phenolic compounds. The analysis identified and quantified caffeic acid, chlorogenic acid, dihydroxybenzoic acid, vanillic acid, myricetin, rutin, and catechin. Representative HPLC-DAD chromatograms showing the separation of these compounds at different wavelengths are provided in Supplementary (Figure S1).

#### 3.3.1. Phenolic Acids

The phenolic acids content (μg/g dw) of cladodes from four *Opuntia* spp. cultivars under different irrigation regimes is shown in Table 1. The concentrations of caffeic, chlorogenic, and vanillic acids increased with greater available water across all investigated nopal cultivars, except for ‘Dalia Roja’ cladodes, where SI stimuli the highest concentrations of dihydroxybenzoic and vanillic acids (*p* < 0.05). Only dihydroxybenzoic acid showed the opposite trend, decreasing as available water increased. Notably, dihydroxybenzoic acid had the highest concentration among all nopal cultivars studied, with the highest levels in ‘Cristalina’ and ‘Amarilla Olorosa’. This phenolic acid has the potential to improve age-related cardiovascular problems, such as hypertension, atherosclerosis, and dyslipidemia [38].

It is known that when a plant has access to more water, it can maintain a more active primary metabolism (photosynthesis, respiration, and growth). These processes ensure a sufficient supply of carbon and energy, along with important substrates for secondary biosynthetic pathways, such as the phenylpropanoid cycle, which produces phenolic acids, including caffeic, chlorogenic, and vanillic acids [39]. Thus, under conditions of sufficient irrigation, secondary metabolism is not limited by a lack of energy or carbon resources, and the synthesis of the necessary precursors that feed the phenylpropanoid pathway could increase. Furthermore, the rise in compounds like caffeic, chlorogenic, and vanillic acids might be connected to their roles in growth and development rather than serving as a defense mechanism against water stress.

#### 3.3.2. Flavonoids

Three flavonoids (catechin, myricetin, and rutin) were identified and quantified in extracts from cladodes of four prickly pear cultivars subjected to different irrigation treatments (Table 2). The results indicated that the concentrations of these metabolites increased significantly under water stress (*p* < 0.05). Among them, myricetin was the most abundant compound across all cladode cultivars, reaching its highest levels in cladodes grown under water deficit (NI). In particular, the ‘Cristalina’ cultivar showed reduced sensitivity to water availability, maintaining myricetin levels relatively unchanged, suggesting an internal regulatory strategy to counter environmental stress, in this case, water deficit. Notably, myricetin is known for its potent free-radical-scavenging activity even at low concentrations [39]; it exhibits antiphotoaging, anticancer, antiplatelet, antihypertensive, immunomodulatory, anti-inflammatory, anti-allergic, and analgesic activities [40].

From a physiological perspective, flavonoids play an important role in plants’ defense mechanisms against water stress. It is known that restricted water availability increases the generation of reactive oxygen species (ROS) [41]. This can activate metabolic pathways, such as the phenylpropanoid pathway, which is responsible for the synthesis of flavonoids and phenolic acids [42]. In this context, the accumulation of myricetin, rutin, and catechin under water-deficit conditions reflects an adaptive response of nopal cladodes to mitigate oxidative damage and maintain cellular integrity.

Decreased phenolic acid content and increased flavonoid content in plants subjected to water-supply restriction have been reported by others. de Lazzari-Almeida et al. [43] observed that the contents of coumarin, chlorogenic acid, and dicaffeoylquinic acid in *Mikania glomerata* decreased under water stress. In another study, concentrations of ellagitannin, gentisic acid, apigenin-7-O-glucoside, and quercetin decreased under water stress, while caffeic acid, apigenin, and acacetin-7-O-glucoside increased in *Dracocephalum moldavica* [44]. Manukyan [45] observed that phenolic acids showed different behavior when plants of different cultivars were subjected to mild, moderate, and severe water stress; species variation was also observed. Thus, bioactive compounds such as polyphenols and flavonoids can increase or decrease in response to the species, type, and intensity of stress [46,47].

Interestingly, cladodes subjected to no irrigation exhibited the highest total flavonoid content despite showing the lowest total polyphenol laves. This apparent contradiction may reflect differential regulation of phenolic subclasses under water deficit. The Folin–Ciocalteu assay estimates total phenolics as a broad group, whereas flavonoids constitute only one subclass of phenolic metabolites. HPLC-DAD analysis revealed that water deficit promoted the accumulation of flavonoids such as catechin, rutin, and myricetin, while several phenolic acids decreased under the same conditions. This response suggests a redistribution rather than a generalized enhancement of phenolic metabolism under drought stress. Water deficit is known to increase reactive oxygen species production, which may preferentially stimulate the synthesis of flavonoids because of their strong antioxidant and protective functions. Consequently, increases in specific flavonoids may occur simultaneously with reductions in other phenolic subclasses, resulting in higher total flavonoid contents but lower total polyphenol values.

### 3.4. Antioxidant Capacity

**Figure 3:**
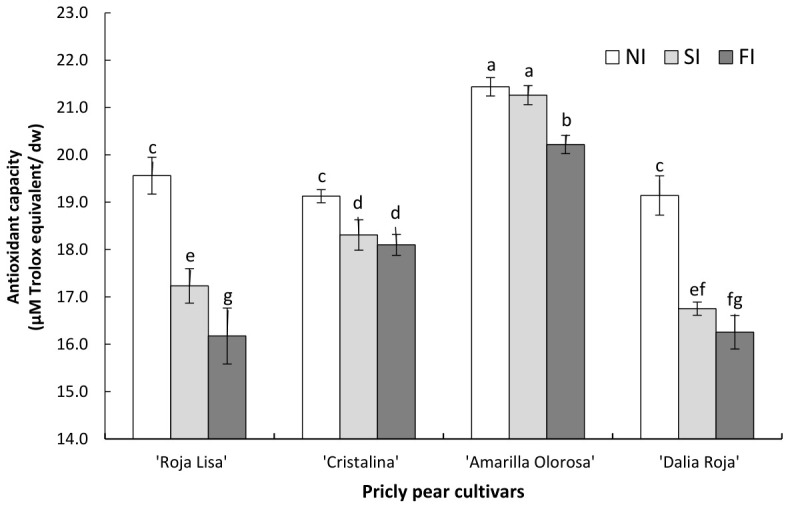
Antioxidant capacity (AC) by ABTS method (mg eq. Trolox/g bs) of prickly pear cladodes undergoing irrigation treatments: no irrigation (NI), supplemental irrigation (SI), and full irrigation (FI) (n = 3). Differences between means were assessed using Fisher’s least significant difference test at *p* ≤ 0.05. Mean values followed by different letters are statistically different. Vertical bars represent the standard deviation of the means.

**Figure 4:**
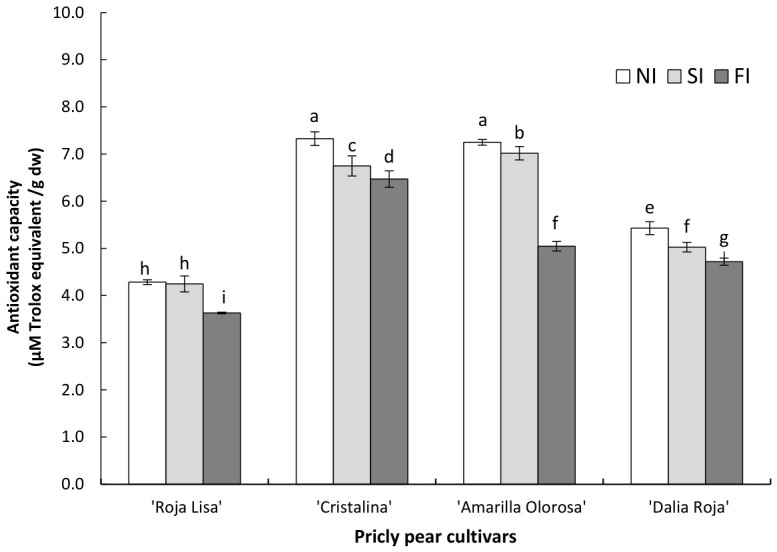
Antioxidant capacity by DPPH method (mg eq. Trolox/g bs) of prickly pear cladodes undergoing irrigation treatments: no irrigation (NI), supplemental irrigation (SI), and full irrigation (FI) (n = 3). Differences between means were assessed using Fisher’s least significant difference test at *p* ≤ 0.05. Mean values followed by different letters are statistically different. Vertical bars represent the standard deviation of the means.

**Figure 5:**
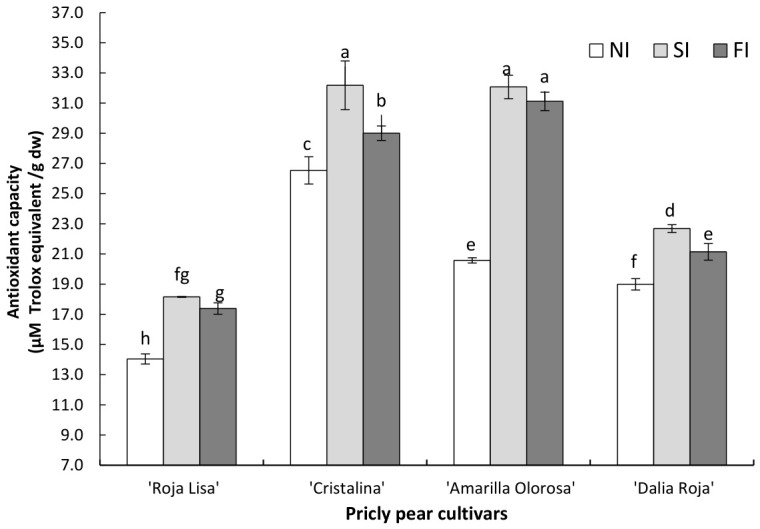
Antioxidant capacity by FRAP method (mg eq. Trolox/g bs) of prickly pear cladodes undergoing irrigation treatments: no irrigation (NI), supplemental irrigation (SI), and full irrigation (FI) (n = 3). Differences between means were assessed using Fisher’s least significant difference test at *p* ≤ 0.05. Mean values followed by different letters are statistically different. Vertical bars represent the standard deviation of the means.

The Antioxidant Capacity (AC) of extracts from cladodes of four *Opuntia* cultivars grown under different irrigation regimes was evaluated using the ABTS, DPPH, and FRAP methods. The AC values, estimated by the ABTS assay, are shown in Figure 3. In general, the nopales subjected to NI exhibited the highest AC (18.38 ± 1.09 µM Trolox equivalents/g dw), followed by those grown under SI. Among the cultivars analyzed, ‘Amarilla Olorosa’ had the highest AC values. Likewise, AC determined using DPPH showed behavior similar to that observed in the ABTS evaluation (Figure 4), confirming the trend of increasing antioxidant capacity under water-deficit conditions. In contrast, FRAP evaluation showed that, in most cases, cultivars subjected to NI had the highest values of Trolox equivalents, followed by those grown under FI (Figure 5). Notably, the ‘Amarilla Olorosa’ cultivar consistently maintained the highest antioxidant capacity under SI and FI conditions, indicating it has the greatest potential in terms of bioactive compounds.

Similar behaviors have been reported in sorghum plants [9], tomato [10,48], the medicinal plant *Melia azedarach* [49], and amaranth leaves [50]. In previous research, water-deficient NI treatments significantly increased plant antioxidant capacity, as measured by the ABTS and DPPH assays. However, no studies have examined the effect of SI on this property.

Likewise, Kaur et al. [51] reported comparable results in chickpea roots. The antioxidant capacity (measured by the FRAP method) remained unchanged in roots under mild water stress, whereas severe drought reduced antioxidant activity. Therefore, decreased antioxidant capacity (FRAP method) in response to reduced water input in cactus plants was associated with reduced total polyphenol content. A strong correlation was found between antioxidant capacity (FRAP) and total polyphenol content (R^2^ = 0.92; *p* = 0.0000). Similarly, Bogale et al. [48] observed a positive correlation between total polyphenol content and FRAP in tomatoes.

Interestingly, a correlation was observed between total flavonoid content and antioxidant capacity measured by the DPPH method (R^2^ = 0.71; *p* = 0.0005). This suggests that reducing water use in cactus plants could promote secondary metabolism and significantly increase flavonoid content, thereby improving antioxidant capacity [10]. Therefore, these results indicate that nopal cladodes grown under SI are a good source of natural antioxidants.

Because bioactive compounds were expressed on a dry weight basis, moisture and dry matter contents were measured to assess the potential influence of tissue water content on the interpretation of results (Supplementary Table S1). Although moisture content differed among cultivars (*p* = 0.036), irrigation treatment did not significantly affect it (*p* = 0.079). Therefore, the differences observed in bioactive compound concentrations among irrigation treatments are unlikely to be explained solely by dilution effects associated with changes in tissue water content. However, we lack additional information to offer a further explanation for these responses.

The phytochemical responses observed in the present study should also be interpreted alongside the agronomic and postharvest responses previously reported under the same experimental conditions and irrigation regimes [16,17,18]. Previous studies demonstrated that supplemental and full irrigation improved plant water status, fruit productivity, fruit quality, and the storability of cactus pear fruit, underscoring the agronomic and commercial relevance of irrigation management in *Opuntia* spp. production systems.

In contrast, the present results indicate that non-irrigated plants exhibited higher flavonoid accumulation and antioxidant capacity, particularly as determined by the DPPH and ABTS assays. This response is consistent with drought-induced activation of secondary metabolism, in which phenolic compounds help mitigate oxidative stress and protect cells under water-deficient conditions.

Taken together, these findings suggest that irrigation management in *Opuntia* spp. affects not only crop productivity and postharvest performance, but also the accumulation of antioxidant metabolites with potential functional and nutraceutical value. Therefore, irrigation strategies should be evaluated in terms of both agronomic performance and phytochemical quality attributes.

One limitation of the present study is that agronomic and postharvest performance variables were not included directly in this manuscript, as those data were previously reported separately within the same experimental framework. Nevertheless, integrating the previously published agronomic findings with the present phytochemical and antioxidant responses provides a broader understanding of the effects of irrigation management in *Opuntia* spp.

## 4. Conclusions

Prickly pear cladodes (*Opuntia* spp.) are a valuable source of bioactive compounds, particularly secondary metabolites known for their antioxidant properties. This study validated that water availability plays a crucial role in influencing the biosynthesis and accumulation of these compounds, in line with the original objective and hypothesis. Specifically, reduced water availability favored the accumulation of flavonoids, which can be interpreted as an adaptive response to oxidative stress induced by water deficit. In contrast, greater water availability promoted the synthesis and accumulation of phenolic acids, likely linked to increased photosynthetic activity. The prominent presence of dihydroxybenzoic acid and myricetin underscores the functional value of nopal, considering the biological properties of these compounds, particularly their potential to prevent cardiovascular alterations and aging.

Likewise, ‘Cristalina’ and ‘Amarilla Olorosa’ cultivars stood out for higher concentrations of bioactive compounds and greater antioxidant capacity under NI, indicating genetic variability in the regulation of secondary metabolism and suggesting differential potential for nutraceutical purposes.

Furthermore, cladodes from plants receiving supplemental irrigation exhibited intermediate to higher concentrations of bioactive compounds and greater antioxidant potential compared to plants grown under moderate water stress or with abundant water. From an agronomic standpoint, this finding indicates that supplemental irrigation can effectively enhance the nutraceutical quality of nopal while preserving yield and promoting more efficient water resource management. Therefore, implementing controlled deficit irrigation could be a sustainable alternative for *Opuntia* production systems and a significant opportunity for the pharmaceutical industry, particularly in arid and semi-arid regions.

## Figures and Tables

**Table 1:** Phenolic acids content (μg/g dw) of *Opuntia* spp. cladodes subjected to irrigation treatments: no irrigation (NI), supplemental irrigation (SI), and full irrigation (FI).

Phenolic Compounds	Treatment	Prickly Pear Cultivars															
		‘Roja Lisa’				‘Cristalina’				‘Amarilla Olorosa’				‘Dalia Roja’			
Caffeic acid (µg g^−1^)	NI	23.63 *	±	0.84	^h^	43.72	±	0.19	^de^	38.61	±	0.03	^f^	32.25	±	0.79	^g^
	SI	29.61	±	2.28	^g^	58.79	±	4.16	^a^	47.03	±	0.96	^cd^	38.71	±	0.22	^f^
	FI	49.41	±	0.98	^bc^	55.76	±	0.07	^a^	51.38	±	4.23	^b^	39.94	±	3.59	^ef^
Chlorogenic acid (µg g^−1^)	NI	115.38	±	7.59	^gh^	467.01	±	0.65	^c^	302.35	±	40.46	^d^	101.68	±	13.94	^h^
	SI	133.10	±	10.61	^gh^	538.04	±	27.45	^b^	479.67	±	4.40	^c^	143.75	±	11.82	^g^
	FI	184.48	±	6.08	^f^	685.38	±	5.53	^a^	493.46	±	1.66	^c^	241.63	±	3.42	^e^
Dihydroxybenzoic acid (µg g^−1^)	NI	400.98	±	19.16	^de^	613.72	±	4.25	^a^	603.27	±	36.33	^a^	497.62	±	1.92	^c^
	SI	359.44	±	3.56	^ef^	534.77	±	8.28	^bc^	551.95	±	15.00	^b^	504.15	±	1.57	^c^
	FI	347.52	±	3.38	^f^	371.20	±	14.35	^ef^	383.39	±	31.00	^ef^	434.06	±	41.04	^d^
Vanillic acid (µg g^−1^)	NI	61.16	±	6.86	^h^	175.40	±	5.20	^c^	115.15	±	4.96	^ef^	125.62	±	4.31	^e^
	SI	79.82	±	4.60	^g^	205.55	±	1.56	^b^	212.15	±	3.66	^b^	155.10	±	5.29	^d^
	FI	102.81	±	5.64	^f^	235.29	±	13.70	^a^	210.85	±	4.35	^b^	127.20	±	5.65	^e^

* By phenolic compound and irrigation treatments, mean values (±standard deviation; n = 3) followed by different letters are statistically different according to Fisher’s least significant difference test at *p* ≤ 0.05.

**Table 2:** Flavonoid content (μg/g dw) of *Opuntia* spp. cladodes subjected to irrigation treatments: no irrigation (NI), supplemental irrigation (SI), and full irrigation (FI).

Phenolic Compound	Treatment	Cultivar															
		‘Roja Lisa’				‘Cristalina’				‘Amarilla Olorosa’				‘Dalia Roja’			
Catechin (µg g^−1^)	NI	123.59	±	6.35 *	^e^	315.25	±	13.01	^b^	408.01	±	7.84	^a^	163.84	±	12.66	^d^
	SI	117.12	±	8.08	^e^	298.58	±	2.06	^bc^	312.88	±	35.04	^bc^	138.18	±	2.72	^de^
	FI	119.52	±	0.11	^e^	285.72	±	5.25	^c^	290.71	±	4.94	^bc^	87.24	±	5.99	^f^
Myricetin (µg g^−1^)	NI	315.28	±	14.30	^f^	1375.34	±	93.62	^c^	1468.83	±	58.52	^b^	1709.67	±	18.06	^a^
	SI	262.42	±	28.39	^f^	1094.40	±	63.25	^d^	853.41	±	21.76	^e^	130.40	±	16.51	^g^
	FI	322.69	±	30.75	^f^	1044.72	±	7.88	^d^	162.29	±	5.23	^g^	163.91	±	0.99	^g^
Rutin (µg g^−1^)	NI	90.67	±	4.87	^gh^	528.54	±	10.14	^a^	295.29	±	28.49	^d^	261.04	±	5.54	^de^
	SI	81.39	±	4.05	^gh^	414.64	±	27.08	^b^	177.76	±	4.16	^f^	242.31	±	12.29	^e^
	FI	59.34	±	2.75	^h^	366.30	±	45.62	^c^	114.81	±	9.77	^g^	227.64	±	3.65	^e^

* By phenolic compound and irrigation treatments, mean values (±standard deviation; n = 3) followed by different letters are statistically different according to Fisher’s least significant difference test at *p* ≤ 0.05.

## Data Availability

The original contributions presented in this study are included in the article/Supplementary Materials. Further inquiries can be directed to the corresponding author(s).

## Supplementary Materials

The following supporting information can be downloaded at: https://www.mdpi.com/article/10.3390/antiox15070787/s1, Table S1: Moisture and dry matter content of mature cladodes from four prickly pear (*Opuntia* spp.) cultivars subjected to different irrigation regimes. Table S2: Calibration curves used for the quantification of phenolic compounds by HPLC-DAD. Figure S1: HPLC-DAD chromatogram of cladodes from the ‘Cristalina’ prickly pear cultivar subjected to supplemental irrigation, showing the separation of polyphenolic compounds at 280 nm (A), 316 nm (B), and 365 nm (C).

## Abbreviations

The following abbreviations are used in this manuscript:

ABTS	2,2-azino-bis (3-ethylbenzthiazoline-6-sulfonic acid)
AC	Antioxidant Capacity
DPPH	2,2-diphenyl-1-picrylhydrazyl
FI	Full Irrigation
FRAP	Ferric Reducing Antioxidant Power
TPC	Total Polyphenolic Content
NI	Non-Irrigated
SI	Supplemental Irrigation
