# Supplementary material for: The Bioactive Compounds and Antioxidant Capacity of Nopal Cladodes (Opuntia spp.) as Influenced by Irrigation
Source: Antioxidants (Basel). 2026 Jun 24;15(7):787. doi: 10.3390/antiox15070787 (PMC13405729; doi:10.3390/antiox15070787)
Supplement: Supplementary file 1 [file antioxidants-15-00787-s001.zip › antioxidants-4367608-supplementary.pdf]

Supplementary material

Table S1. Moisture and dry matter content of mature cladodes from four prickly pear (*Opuntia* spp.) cultivars subjected to different irrigation regimes.

Cultivar	Irrigation treatment	Moisture (%)	Dry matter (%)
'Roja Lisa'	FI	85.03 ± 1.11	14.97 ± 1.11
	NI	81.46 ± 1.73	18.54 ± 1.73
	SI	84.27 ± 4.40	15.73 ± 4.40
'Cristalina'	FI	84.99 ± 0.09	15.01 ± 0.09
	NI	85.28 ± 1.92	14.72 ± 1.92
	SI	83.34 ± 1.77	16.66 ± 1.77
'Amarilla Olorosa'	FI	83.80 ± 0.41	16.20 ± 0.41
	NI	83.10 ± 1.47	16.90 ± 1.47
	SI	84.27 ± 1.49	15.73 ± 1.49
'Dalia Roja'	FI	85.45 ± 0.58	14.55 ± 0.58
	NI	82.84 ± 2.21	17.16 ± 2.21
	SI	87.96 ± 3.24	12.04 ± 3.24

Values are reported as mean ± standard deviation (n = 3). FI, Full irrigation; SI, Supplemental irrigation; NI, No irrigation. Moisture content was significantly affected by cultivar (p = 0.036), whereas irrigation treatment (p = 0.079) and the cultivar × irrigation treatment interaction (p = 0.764) were not significant.

Table S2. Calibration curves used for the quantification of phenolic compounds by HPLC-DAD.

Compound	Concentration range (mg L^{-1})	Regression equation	R^2
Caffeic acid	2.5–50	y = 224.67x − 82.96	0.9996
Chlorogenic acid	10–200	y = 11.951x + 29.436	0.9989
Dihydroxybenzoic acid	5–50	y = 59.604x − 231.61	0.9966
Vanillic acid	5–200	y = 52.39x − 98.038	0.9995
Catechin	5–200	y = 29.015x + 20.113	0.9984
Myricetin	2.5–50	y = 139.13x − 130.96	0.9998
Rutin	5–100	y = 71.108x − 56.288	0.9991

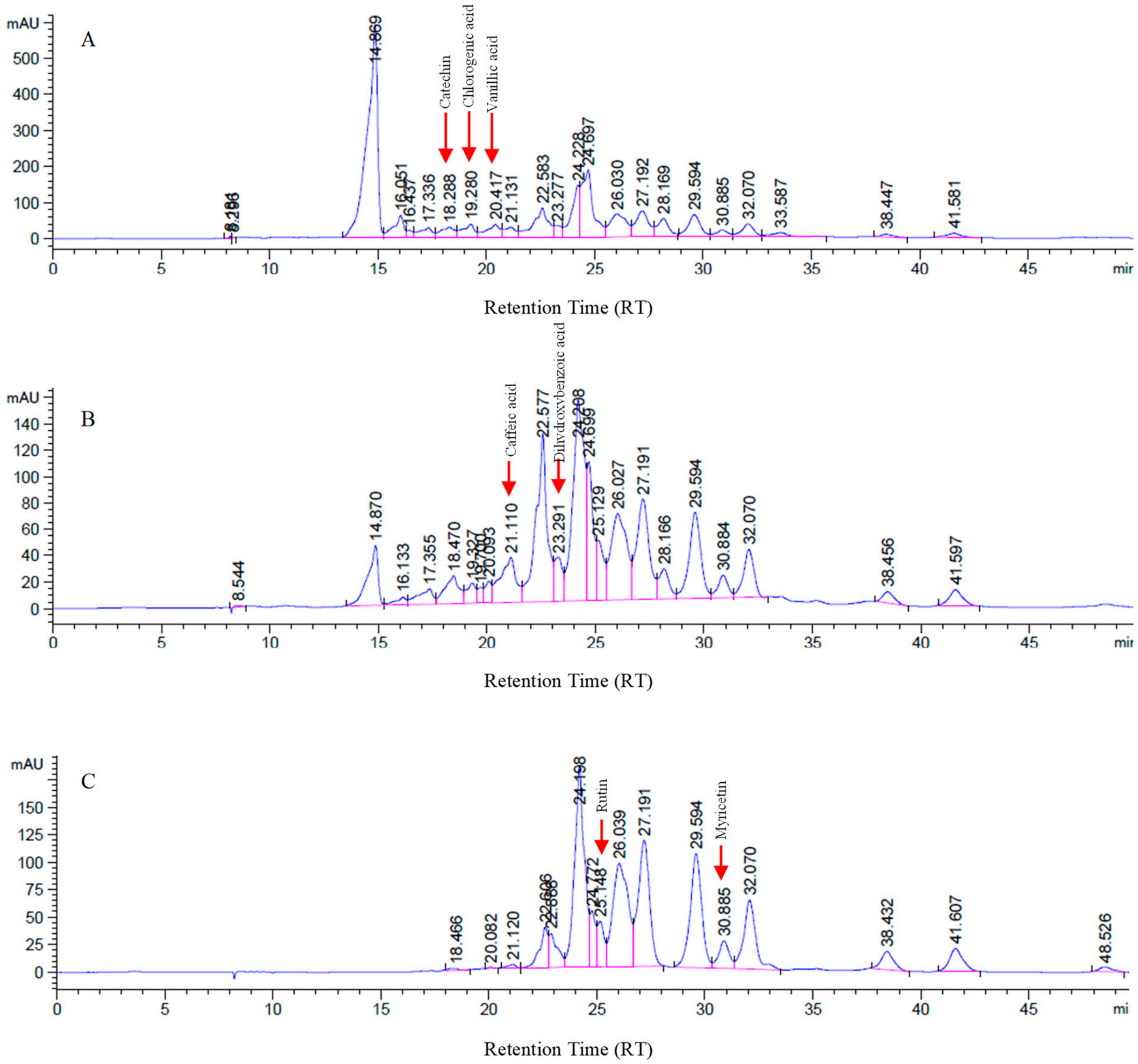

Figure S1. HPLC-DAD chromatogram of cladodes from the 'Cristalina' prickly pear cultivar subjected to supplemental irrigation, showing the separation of polyphenolic compounds at 280 nm (A), 316 nm (B), and 365 nm (C).
